# Community Structure of Arbuscular Mycorrhizal Fungi in Soils of Switchgrass Harvested for Bioenergy

**DOI:** 10.1128/AEM.00880-20

**Published:** 2020-09-17

**Authors:** Alden C. Dirks, Randall D. Jackson

**Affiliations:** a Department of Agronomy, University of Wisconsin—Madison, Madison, Wisconsin, USA; University of Toronto

**Keywords:** DADA2, Glomeromycotina, PacBio Sequel SMRT sequencing, amplicon sequence variants, fungal metabarcoding

## Abstract

Learning more about the biodiversity and composition of arbuscular mycorrhizal fungi (AMF) under alternative agricultural management scenarios may be important to the sustainable intensification of switchgrass grown as a bioenergy crop. Using PacBio single-molecule sequencing and taxonomic resolution to the level of amplicon sequence variant (ASV), we assessed the effects of nitrogen amendment on AMF associating with switchgrass and explored relationships between AMF and switchgrass yield across three sites of various productivities in Wisconsin. Nitrogen amendment had little effect on AMF diversity metrics or community composition. While AMF ASV diversity was not correlated with switchgrass yield, AMF family richness and switchgrass yield had a strong, positive relationship at one of our three sites. Each of our sites was dominated by unique ASVs of the species Paraglomus brasilianum, indicating regional segregation of AMF at the intraspecific level. Our molecular biodiversity survey identified putative core members of the switchgrass microbiome, as well as novel clades of AMF, especially in the order Paraglomerales and the genus Nanoglomus. Furthermore, our phylogenies unite the cosmopolitan, soil-inhabiting clade deemed GS24 with Pervetustaceae, an enigmatic family prevalent in stressful environments. Future studies should isolate and characterize the novel genetic diversity found in switchgrass agroecosystems and explore the potential yield benefits of AMF richness.

**IMPORTANCE** We assessed the different species of beneficial fungi living in agricultural fields of switchgrass, a large grass grown for biofuels, using high-resolution DNA sequencing. Contrary to our expectations, the fungi were not greatly affected by fertilization. However, we found a positive relationship between plant productivity and the number of families of beneficial fungi at one site. Furthermore, we sequenced many species that could not be identified with existing reference databases. One group of fungi was highlighted in an earlier study for being widely distributed but of unknown taxonomy. We discovered that this group belonged to a family called Pervetustaceae, which may benefit switchgrass in stressful environments. To produce higher-yielding switchgrass in a more sustainable manner, it could help to study these undescribed fungi and the ways in which they may contribute to greater switchgrass yield in the absence of fertilization.

## Abstract (precis)

We assessed the different species of beneficial fungi living in agricultural fields of switchgrass, a large grass grown for biofuels, using high-resolution DNA sequencing. Contrary to our expectations, the fungi were not greatly affected by fertilization. However, we found a positive relationship between plant productivity and the number of families of beneficial fungi at one site. Furthermore, we sequenced many species that could not be identified with existing reference databases. One group of fungi was highlighted in an earlier study for being widely distributed but of unknown taxonomy. We discovered that this group belonged to a family called Pervetustaceae, which may benefit switchgrass in stressful environments. To produce higher-yielding switchgrass in a more sustainable manner, it could help to study these undescribed fungi and the ways in which they may contribute to greater switchgrass yield in the absence of fertilization.

## INTRODUCTION

Arbuscular mycorrhizal fungi (AMF) are ubiquitous soil-dwelling organisms from the phylum Mucoromycota, subphylum Glomeromycotina, that form symbiotic relationships with upwards of 80% of vascular plant families (1, 2). AMF can confer many benefits to their hosts, including pathogen protection, drought tolerance, and water acquisition, but principal among them is the uptake of mineral nutrients. The AMF mycelium mines nitrogen (N), phosphorus (P), and other nutrients from the soil and transfers them to host plants via intraradical structures called arbuscules. In exchange, host plants share photosynthetically fixed carbon (C) with the AMF (3, 4). In recent years, global interest in sustainable agricultural intensification has brought AMF to the forefront as natural biofertilizers to offset synthetic fertilizer application. However, knowledge gaps concerning the effects of agricultural management (e.g., N fertilization) on AMF hinder farmers’ ability to leverage “mycorrhizal technology” for sustainability and productivity goals (5–7).

To better understand the effects of N addition on AMF biodiversity and composition, we assayed the mycorrhizal communities associating with switchgrass (Panicum virgatum L.) in N-amended and unamended stands in Wisconsin. Switchgrass, a warm-season plant endemic to the tallgrass prairies of North America, is cultivated as a cellulosic biofuel feedstock and forage crop for cattle (8). Switchgrass agroecosystems can provide a number of important ecosystem services, such as soil and nutrient retention, that make it an attractive option for low-carbon-economy agricultural landscapes (9). However, switchgrass cultivation is hampered in part by the highly context-dependent relationship between N addition and plant growth (10). While a global meta-analysis reported that N fertilization significantly increases switchgrass growth (11), N amendment did not increase switchgrass aboveground net primary productivity compared to that in unamended plots in a 2-year agronomic trial in Wisconsin (12). Similar results were found by Jach-Smith and Jackson (13), Ruan et al. (14), Wang et al. (15), and Emery et al. (16). Duran et al. (12) hypothesized that this variation in switchgrass response is a function of microbial N immobilization. Since switchgrass is highly mycorrhiza-dependent (17) and AMF are sensitive to soil stoichiometry and agricultural intensification (18–20), increasing our knowledge of AMF in these agroecosystems could help explain variation in switchgrass response to N addition (6).

While switchgrass generally benefits from N supplied by AMF (21), exogenous N inputs can decrease the abundance and functioning of AMF, potentially negating their symbiotic benefits (22). Jach-Smith and Jackson (23) found that N amendment decreased AMF root colonization of switchgrass and allocation of resources to nutrient transfer structures. In a follow-up study, Jach-Smith and Jackson (24) showed that N addition effectively replaced N that otherwise would have been supplied to switchgrass by AMF. Conversely, in a microcosm experiment, elevating N did not influence mycorrhizally mediated N uptake and transfer to switchgrass (25). Moreover, Emery et al. (26) found that N amendment did not affect AMF root colonization of switchgrass or extraradical hyphae, but it slightly decreased AMF operational taxonomic unit richness and the Shannon diversity index. Considering these conflicting findings, it is difficult to discern a general pattern in the effects of N addition on AMF associating with switchgrass (27).

Part of the uncertainty around the effects of N addition on AMF and switchgrass productivity could stem from the limited taxonomic resolution of traditional methods used to characterize AMF, which fail to detect certain clades while masking the extensive functional diversity present among AMF after over 500 million years of evolution (28). For example, AMF staining procedures to measure root colonization and nutrient transfer structures do not detect members of the order Paraglomerales, which have weakly staining or completely nonstaining tissues (29, 30). Furthermore, morphological metrics have no inherent meaning for plant performance, given that AMF species exist along a symbiotic spectrum ranging from mutualism to parasitism (31, 32). In fact, alternative strains within a given AMF species can result in completely different outcomes for plant performance (33–35). In addition, the 16:1ω5cis fatty acid biomarker commonly used to measure AMF abundance in soils (36, 37) is only present in miniscule quantities or is completely absent from species in the Paraglomeraceae and Gigasporaceae (38). On the other hand, fatty acid indicators for these two families (e.g., 16:1ω7cis, 18:1ω9cis, 20:1ω9cis) are typically treated as biomarkers for saprotrophic fungi and Gram-negative bacteria, conflating the abundance of multiple guilds (38–40). Finally, AMF metabarcoding of the small-subunit (SSU) ribosomal DNA (rDNA) gene region with short-read sequencing platforms does not discriminate AMF to the species level (27, 41, 42). This problem is compounded when operational taxonomic units are clustered at 97% similarity, a conservative threshold and arbitrary proxy for species-level delimitation of filamentous fungi (43, 44).

In the context of a long-term, replicated bioenergy cropping systems experiment, we conducted a metabarcoding study of AMF using PacBio Sequel single-molecule real-time (SMRT) sequencing (see Note S1 in the supplemental material) and characterized the communities to the level of amplicon sequence variant (ASV) for a high-resolution investigation of the effects of N addition on AMF composition (41). PacBio offers comparatively long sequence reads spanning conserved and highly variable regions of rDNA for an unprecedented opportunity to investigate AMF at a fine taxonomic resolution (42). ASVs are exact, error-free sequences and thus serve as biologically meaningful representatives of AMF genotypes, revealing intraspecific variation and strains of interest for bioprospecting (9, 43). Our study employed PacBio Sequel SMRT sequencing with ASVs in the analysis of AMF communities and is one of only a handful to use PacBio for AMF metabarcoding (18, 41, 45–47). We asked the following questions: (i) does long-term N amendment affect AMF ASV diversity or community structure in Wisconsin switchgrass agroecosystems? and (ii) does AMF diversity explain variation in switchgrass yield? We hypothesized that N amendment would decrease AMF diversity (27, 48) and that AMF diversity would be correlated with switchgrass yield because of its positive linkage to ecosystem functioning (3).

## RESULTS

### Taxonomy and phylogeny of ASVs recovered from switchgrass agroecosystems.

**FIG 1:**
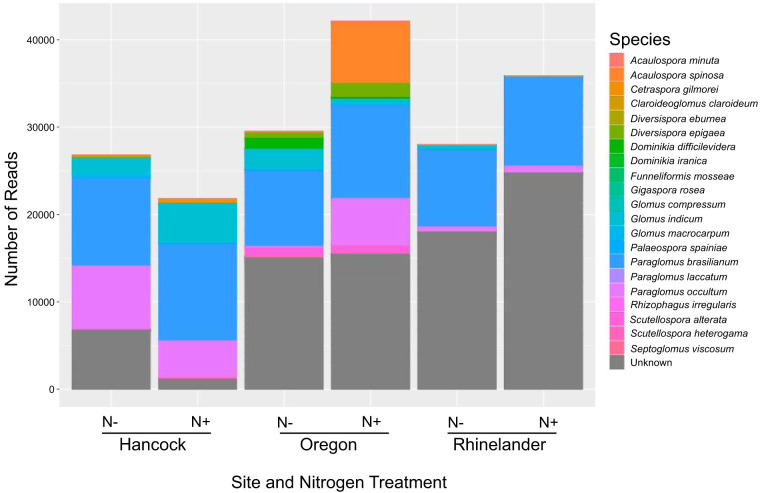
Summary of taxonomic distribution of ASVs according to the UNITE ITS fungal database by site and nitrogen treatment. N^−^ refers to unamended experimental units (0 kg N · ha^−1^), and N^+^ refers to the amended experimental units (56 kg N · ha^−1^).

A total of 190,700 high-quality, nonchimeric amplicons clustered into 436 ASVs (for a complete breakdown of the number of sequences remaining after each bioinformatics processing step, see Table S2 in the supplemental material). Rarefaction curves of ASV accumulation versus sample size reached a clear asymptote for every experimental unit, indicating relatively complete sampling of AMF diversity (see Fig. S2 in the supplemental material). According to UNITE taxonomic assignment, the 436 ASVs were spread across Glomeromycotina, representing all four orders in the subphylum. In addition, 7 of 12 families, 13 of 41 genera, and 21 of 334 species that are currently described in Glomeromycotina were recovered. However, almost half of the ASVs (211; 48.4%) could not be assigned to a species hypothesis (Fig. 1), and more than a quarter (119; 27.3%) could not be assigned to the level of genus. While all ASVs were placed in Glomeromycotina, a few (24; 5.5%) were unassignable even at the order level. Manual taxonomic assignment according to phylogeny resulted in the representation of all four orders, 9 of 12 families, 20 of 41 genera, and 17 of 334 species currently described in Glomeromycotina, as well as an additional 36 well-supported clades of unknown specific identity (see Fig. S3 and S4 in the supplemental material).

Comparing UNITE taxonomic assignments to our Glomeromycotina phylogenetic tree (Fig. S3 and S4) indicated that certain clades, while described in the literature, were not represented in the UNITE database and thus taxonomic assignment of related ASVs consistently failed. Of the 24 ASVs that could not be assigned to any order, 18 were resolved to a Glomerales clade encompassing the recently described species Nanoglomus plukenetiae (49) and the remaining six to the *Pervetustaceae* clade in Paraglomerales (29). Furthermore, eight ASVs were closely related to described species in Paraglomeraceae or *Pervetustaceae* in Paraglomerales but were not assigned to any family. Of the 119 ASVs that were not assigned a generic rank, 44 belonged to the clade encompassing *Nanoglomus plukenetiae*. This nonexhaustive exploration of failures in taxonomic assignment showcases both the biological (e.g., myriad undescribed taxa in the *Nanoglomus plukenetiae sensu lato* clade) and artificial (e.g., incomplete data curation of Paraglomerales) issues that hinder accurate identification of AMF environmental sequences using even curated reference databases.

**FIG 2:**
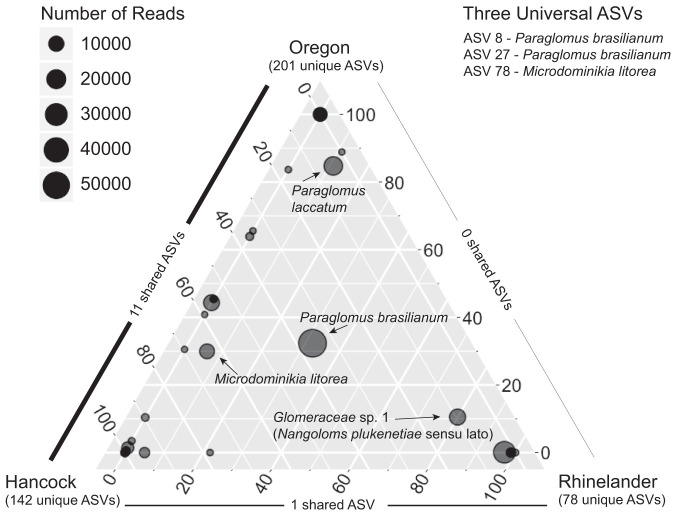
Ternary plot showing the site distributions for 53 AMF species-level clades determined by LSU rDNA phylogenies. Taxa present with >4% relative abundance at all three sites are labeled with their names. The size of points corresponds to the total sequence abundance for that taxon across all three sites. Most taxa are only present at one site or are rarely present at the others. As a result, most points are positioned at the tips of the ternary plot. The number of unique ASVs for a given site and the number of shared ASVs between two sites are indicated along the axes on the outside of the ternary plot. Three ASVs, namely ASVs 8, 27, and 78, were found at all three sites.

A few clades stood out for their high sequence abundance or consistent presence across the experimental units, which suggests a role in the core microbiome of switchgrass (Fig. 2) (50). The previously mentioned clade encompassing *Nanoglomus plukenetiae* included ASVs with some of the greatest absolute abundances (ASVs 1, 4, and 9). While no ASV belonging to this clade occurred at more than one site, representatives of the *Nanoglomus plukenetiae sensu lato* clade were present in both N-amended and control plots at all three sites (Fig. S3 and S4). In total, 64 of the 436 ASVs (∼15%) belonged to this diverse but poorly understood clade, which may represent various undescribed species in the genus Nanoglomus or new genera within Glomeraceae. Within Paraglomerales, 73 ASVs (∼17%) had high LSU sequence similarity to Paraglomus brasilianum and 51 more (∼12%) to Paraglomus laccatum. Since ASVs belonging to these species also occurred at all three sites (Fig. S3 and S4), *Paraglomus* spp. appear to be important members of the core switchgrass microbiome as well. Of the 436 ASVs recovered in this study, only three ASVs occurred at all three sites, two of which belonged to *Paraglomus brasilianum* (ASVs 8 and 27); the last, ASV 78, belonged to Microdominikia litorea, another potential member of the core switchgrass microbiome (Fig. 2).

**FIG 3:**
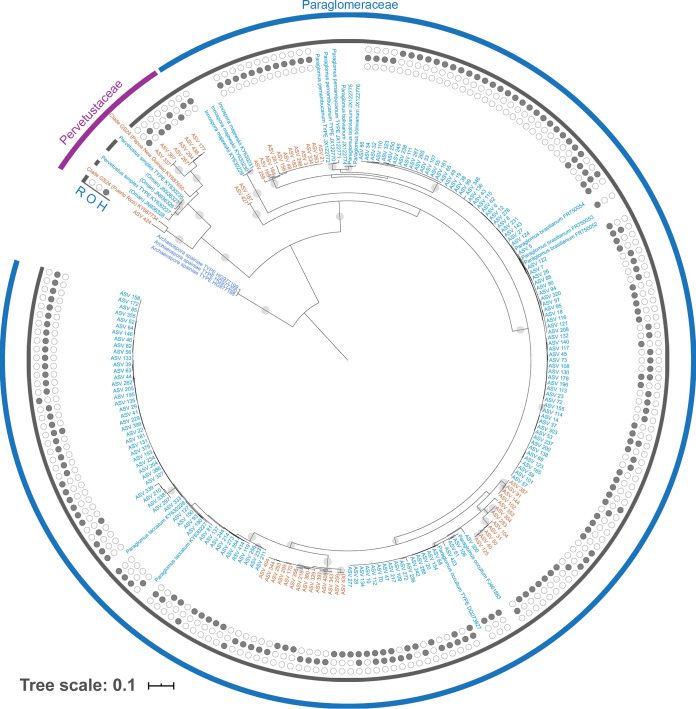
Phylogenetic tree of Paraglomerales reference taxa and recovered ASVs, as well as representatives of the previously unknown clade GS24 (51) with Archaeospora spainiae (Archaeosporales) as the outgroup. Site occurrence is indicated by the filled circles on the outside of the tree. “H” refers to Hancock (inside circles), “O” to Oregon (middle circles), and “R” to Rhinelander (outside circles) sites. Bootstrap support of ≥0.75 is indicated by gray dots on the branches, with the size of the dots corresponding to relative bootstrap support. Teal text indicates ASVs or reference taxa with a clear taxonomic affinity, while orange text indicates ASVs that could not be assigned to a species name based on available phylogenetic information.

In addition to the Nanoglomus plukenetiae *sensu lato* clade, some of the less-abundant clades identified in this study might be undescribed lineages of AMF. Our phylogenetic analysis showed that the unknown, worldwide, soil-inhabiting clade deemed GS24 (51) corresponds to the family *Pervetustaceae* in Paraglomerales, which contains one species, Pervetustus simplex, described in 2017 from the deserts of Oman (29). We recovered seven ASVs in three clades belonging to this enigmatic family, two of which were well supported (Fig. 3). These ASVs were most prevalent in the sandy soils of our Hancock site, indicating that they may confer some degree of stress tolerance to switchgrass under water-limiting or nutrient-poor conditions (50). Finally, two ASVs, ASVs 187 and 427, were phylogenetically resolved as basal to the rest of the *Paraglomeraceae*, suggesting that a new genus or a new family in Paraglomerales might be necessary to accommodate this newfound genetic diversity (Fig. 3).

### Effects of nitrogen amendment on switchgrass yield and soil variables.

**FIG 4:**
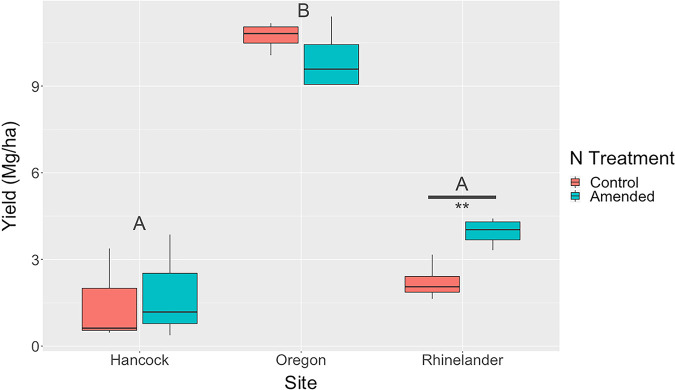
Boxplots of switchgrass yield at three sites under N addition (amended) or no N addition (control) treatments. The boxplots show the minimum, median, maximum, and interquartile range of the data. The letters above the boxplots indicate statistical difference of switchgrass yield between sites, with different letters representing statistically significant differences (*P < *0.05). The horizontal bar above the boxplots indicates statistical difference of switchgrass yield between treatments within sites; two asterisks denote a significant difference (*P < *0.01) in switchgrass yield between amended and control plots at the Rhinelander site.

**FIG 5:**
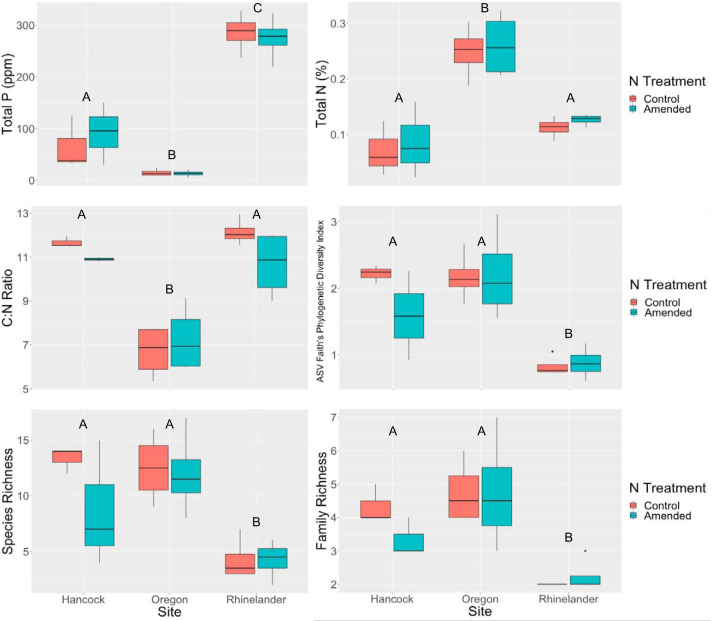
Boxplots of soil variables at three sites under N addition (amended) or no N addition (control) treatments. The boxplots show the minimum, median, maximum, and interquartile range of the data. The letters above the boxplots indicate statistical difference of soil variables between sites, with different letters representing statistically significant differences (*P < *0.05). No soil variables differed significantly between the two N treatments within a site.

**FIG 6:**
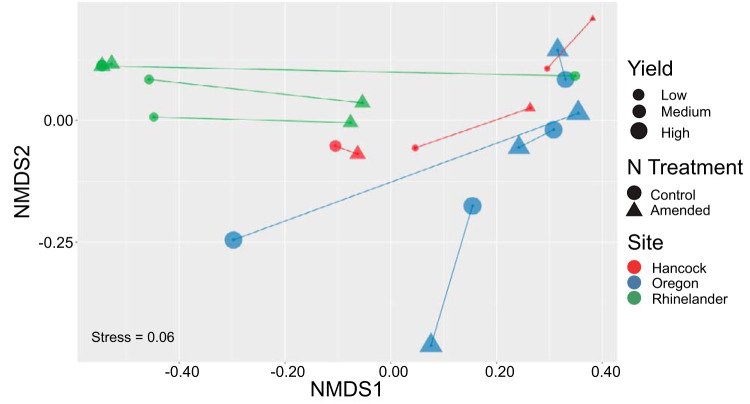
Nonmetric multidimensional scaling (NMDS) of UniFrac beta dissimilarity. Each point corresponds to the AMF community of an experimental unit. The size of points represents switchgrass yield, the shape of points corresponds to N treatment, and the color of points indicates site. Paired experimental units are linked by lines.

The effect of N amendment on square-root-transformed switchgrass yield depended on site (site × N interaction, *F*_2,8_ = 14.04, *P = *0.002; Fig. 4). At the Rhinelander site, N amendment enhanced switchgrass yield compared to that of unamended control plots (*t*_8_ = 5.93, *P < *0.01) but had no significant effect at the Oregon (*t*_8_ = −1.52, *P = *0.66) or Hancock (*t*_8_ = 1.21, *P = *0.82) sites. Log-transformed total P, total N, C:N ratio, ASV Faith’s phylogenetic index, species richness, and square-root-transformed family richness differed significantly by site (Fig. 5), but no soil variables were significantly affected by nitrogen amendment (Table 1). Permutational analysis of variance (PERMANOVA) results indicated that ASV UniFrac community dissimilarity was not significantly affected by N treatment (*F*_1,20_ = 0.23276, *P > *0.80; Fig. 6). However, community dissimilarity was significantly correlated with dissimilarity in soil total N (*r* = 0.12, *P = *0.004).

**TABLE 1:** Linear mixed effects regression models type III ANOVA results^*a*^

Response variable	Site		Nitrogen		Site × nitrogen	
	*F*_(2,8)_	*P*	*F*_(1,8)_	*P*	*F*_(2,8)_	*P*
Square root-transformed switchgrass yield (Mg/ha)	33.39	**<0.0001**	10.10	0.01	14.04	**0.002**
pH	6.11	0.03	0.04	0.85	0.29	0.76
Cation exchange capacity (cmol_c_/kg)	8.68	0.01	0.34	0.58	0.05	0.95
Log-transformed total P (ppm)	40.63	**<0.0001**	0.26	0.63	2.11	0.18
Total C (%)	4.85	0.04	4.21	0.07	1.80	0.23
Total N (%)	17.08	**0.001**	2.39	0.16	0.01	0.99
C:N ratio	27.01	**0.0003**	2.89	0.13	3.53	0.08
ASV richness	8.67	0.01	0.35	0.57	2.44	0.15
ASV Shannon diversity index	3.68	0.07	1.32	0.28	0.96	0.42
ASV Simpson’s diversity index	3.90	0.07	0.54	0.48	0.09	0.91
ASV Pielou’s evenness index	0.62	0.56	0.59	0.47	0.04	0.96
ASV Faith’s phylogenetic diversity index	20.97	**0.0007**	0.99	0.35	1.32	0.32
ASV phylogenetic mean pairwise distance	3.88	0.07	1.88	0.21	3.11	0.10
Species richness	14.65	**0.002**	1.62	0.24	1.09	0.38
Species Shannon diversity index	3.01	0.11	5.82	0.04	3.44	0.08
Species Simpson’s diversity index	1.29	0.33	2.32	0.17	3.33	0.09
Species Pielou’s evenness index	0.09	0.92	4.32	0.07	3.44	0.08
Genus richness	10.81	0.005	1.18	0.31	2.12	0.18
Genus Shannon diversity index	0.86	0.46	1.23	0.30	3.58	0.08
Genus Simpson’s diversity index	0.24	0.79	0.29	0.60	3.83	0.07
Genus Pielou’s evenness index	0.21	0.82	0.44	0.53	0.63	0.56
Family richness	14.68	**0.002**	1.02	0.34	2.22	0.17
Family Shannon diversity index	0.89	0.45	0.24	0.64	3.56	0.08
Family Simpson’s diversity index	0.30	0.75	0.004	0.95	3.25	0.09
Family Pielou’s evenness index	0.26	0.78	0.28	0.61	1.49	0.28

a Significant variables at the Bonferroni-corrected threshold of *P *< 0.002 are indicated in bold. ANOVA, analysis of variance.

### Relationship between soil variables, switchgrass yield, and indicator taxa.

**FIG 7:**
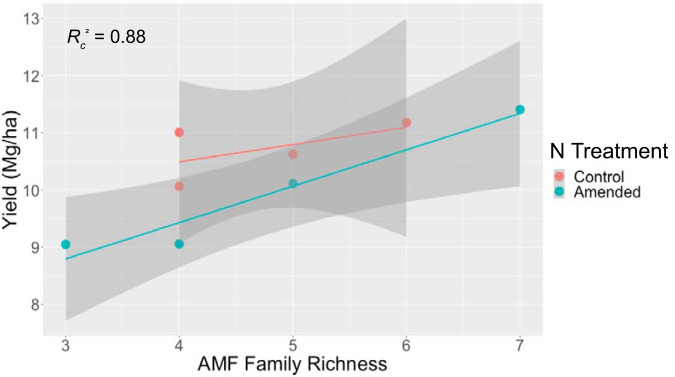
Scatterplot showing the relationship between AMF family richness and N treatment on switchgrass yield at the Oregon site. Orange points correspond to experimental units under no N amendment (control) and teal points correspond to experimental units under N amendment (amended) conditions. The lines correspond to best-fitting linear regressions. The gray shading around the regression lines is the 95% confidence intervals of the regressions.

No metric of AMF ASV diversity was a significant predictor of switchgrass yield at any site in linear mixed-effects regression models including N treatment. At the family level, AMF richness was a marginally significant predictor of yield at the Oregon site (*F*_1,4.92_ = 21.3, *P = *0.006, conditional coefficient of determination [*R_c_*^2^] = 0.88); every additional family represented in the AMF community at the Oregon site was associated with an increase in 0.61 Mg · ha^−1^ of switchgrass yield (Fig. 7). There was also a significant relationship between switchgrass yield and cation exchange capacity at the Hancock site (*F*_1,3_ = 157.3865, *P* = 0.001). Holding N addition constant, every unit increase in cation exchange capacity was associated with an increase of 0.38 Mg · ha^−1^ of switchgrass.

No taxa were indicators of N treatment, whether analyzed at the level of ASV, species, genus, family, or order. A total of 13 ASVs were indicators of site, occurring exclusively at their respective sites and in 50% or more of plots at that site. Three *Paraglomus brasilianum* ASVs (ASVs 5, 18, and 114) and two *Microdominikia litorea* ASVs (ASVs 98 and 139) were indicators of the Hancock site (*P < *0.02). Three *Paraglomus brasilianum* ASVs (ASVs 6, 12, and 65) were also indicators of the Rhinelander site, as well as one *Nanoglomus plukenetiae sensu lato* ASV (ASV 1; *P ≤ *0.02). Two Paraglomus laccatum ASVs (ASVs 41 and 63), one Scutellospora calospora ASV (ASV 149), and an unknown Rhizoglomus species (ASV 186) were indicators of the Oregon site (*P ≤ *0.03). At the familial level, *Pervetustaceae* was an indicator at the Hancock site, occurring in half of the plots and almost exclusively at this site (*P < *0.05).

## DISCUSSION

### N addition did not affect AMF ASV diversity or composition.

Contrary to our expectations, we found no effect of N addition on AMF ASV diversity or community composition. No consensus exists on how N enrichment affects AMF diversity, despite the considerable amount of research this topic has received (27). It is difficult to compare the effects of N addition across studies given the different methodologies employed to assess AMF diversity, in particular metabarcoding studies versus spore-based studies. We observed that the most frequently recovered ASVs from switchgrass soils were primarily those with small spores (e.g., AMF belonging to the genera *Dominikia*, *Nanoglomus*, Microkamienskia, *Paraglomus*, and *Rhizoglomus*) or species that sporulate rarely or only seasonally (e.g., Acaulospora spp.), which would have made morphology-based detection and classification extremely difficult even for expert taxonomists (52). In addition, we detected numerous undescribed lineages of AMF of unknown taxonomic diversity—for example, it is unclear how many species were represented in the *Nanoglomus plukenetiae sensu lato* clade detected in this study—that would have made accurate morphotyping possible only after extensive taxonomic research concerning the undescribed genetic diversity found in switchgrass agroecosystems.

In addition to limitations in AMF taxonomy, a robust predictive framework concerning the effects of N addition on AMF is elusive considering the number of variables that might play a role in the context dependency of AMF response to N addition. These include plant community diversity, host photosynthetic pathway, time of the year, initial diversity of the AMF community, and baseline soil nutrients (27, 53). Given that Antoninka et al. (48) observed decreased AMF diversity in monocultures but not in polycultures, and that Egerton-Warburton et al. (54) reported decreased diversity of AMF communities associated with warm-season grasses but not with cool-season grasses, we expected N addition to negatively affect AMF diversity in the context of switchgrass monocultures. Theories of soil resource stoichiometry predict that AMF are sensitive to ratios of available N and P (19). The N application rate used in this study had no significant effect on soil N or P at any site, so the fact that we did not observe changes in ASV diversity with N amendment may be consistent with resource stoichiometry theory. Although our rate of N amendment matched estimates for switchgrass harvest removal (15), this is only about half of the recommended N application rate (55, 56). At higher application rates of N, shifts in soil resource stoichiometry could be more apparent, with subsequent effects on AMF diversity (57). Finally, in a metanalysis of the effects of N addition on AMF Shannon diversity, short-term (<5-year) fertilization studies had no effect on AMF diversity (57). We sampled AMF communities after 4 years of N addition, which may not have been sufficient time to observe consistent changes in AMF.

Previous studies have reported shifts in AMF community composition without changes in alpha diversity (57). N enrichment can affect AMF community structure via competitive effects where AMF compete with each other for host C, or via selective effects, where AMF species have different sensitivities to agricultural inputs (53). N amendment had no discernible effect on AMF community structure or on soil N in the time frame of our study, but the correlation between AMF community structure and soil N within sites suggests that competitive or selective effects were at play independent of N treatment. Treseder et al. (58) found that AMF with extensive extraradical hyphae (e.g., *Gigasporaceae*) had low tolerance of elevated soil N, but AMF with greater allocation to intraradical structures (e.g., Glomus spp.) were more prevalent in N-enriched environments. We recovered relatively few *Gigasporaceae* AMF, and little functional information exists on other taxa prevalent in our study (e.g., Paraglomerales), limiting our ability to determine whether community structure in relation to soil N was a function of morphology.

### Positive correlation trend between yield and AMF family richness at most productive site.

We found no universal relationships between AMF metrics and switchgrass yield at the ASV level. Indeed, improved taxonomic resolution may not allow one to detect relationships between AMF and plant productivity if fine-scale taxonomic groupings of AMF are not good proxies for functional trait differences. However, at the family level in our most productive site (Oregon), we detected a relationship between switchgrass yield and AMF family richness. Along with N treatment and plot, these variables could account for 88% of the variation in switchgrass yield. In another bioenergy study on productive soils, Emery et al. (59) did not observe a correlation between switchgrass yield and AMF diversity, but they did not analyze AMF diversity at the family level. According to Chagnon et al. (60), AMF functional traits are shared at the family level. Thus, with greater richness of AMF families, switchgrass may have benefitted from greater diversity as a result of AMF functional complementarity (e.g., P acquisition, plant pathogen protection, and abiotic stress tolerance, among others). This result is consistent with a meta-analysis by Yang et al. (61), which found that plant performance was positively promoted by AMF family richness but not species richness or phylogenetic diversity. In our study, all experimental units at the Oregon site contained ASVs from Glomeraceae and Paraglomeraceae. Interestingly, only the two most productive experimental units contained ASVs from *Pervetustaceae*, a recently described family with worldwide occurrence for which little functional information exists.

Future research should explore the generality of these results and the potential yield benefits of actively augmenting AMF family richness via spore inoculations. House and Bever (62) demonstrated that the establishment of Andropogon gerardii, a tallgrass prairie plant closely related to switchgrass, benefited from inoculation with seven regionally adapted AMF morphotypes, suggesting that a small number of diverse AMF species is sufficient to observe beneficial effects of AMF inoculation on plant establishment. Accordingly, inoculations of switchgrass agroecosystems that include regionally adapted AMF from multiple families might result in benefits not only to switchgrass yield but also to switchgrass establishment. These benefits could make switchgrass a more viable crop for farmers to grow, as establishment and early-season productivity are agronomic impediments to its widespread cultivation (15, 63). In inoculation experiments, we recommend investigators start with the putative core AMF of the switchgrass microbiome and supplement the microbiome with representatives from the families present in the highest-yielding plots of the Oregon site.

### Switchgrass soil microbiome dominated by poorly studied taxa.

Our understanding of AMF is biased toward those taxa that are most prevalent in disturbed habitats (64). Concerted efforts to understand uncultured fungi in less disturbed environments could benefit perennial agriculture as a whole, given that these agroecosystems might harbor AMF communities that are more similar to those of nonagricultural ecosystems. In addition to the numerous undescribed clades identified in this study, little is known about the ecology of the putative core AMF of the switchgrass microbiome. *Microdominikia litorea* was described in 2018 in association with Xanthium spinosum, a spiny plant growing on Mediterranean sand dunes (65). When we compared our ASVs of *Microdominikia litorea* to environmental sequences in GenBank, only two isolates from grasslands of northern China were similar (GenBank accession numbers KF836940 and KF936961; 100% query cover, >97.5% identical). *Nanoglomus plukenetiae* was only recently described from the Amazonian lowlands of Peru in association with the Inca nut, Plukenetia volubilis (49). Deposited sequences from an unpublished switchgrass study at the Konza Prairie Biological Station near Manhattan, Kansas, were >99% identical to that of our *Glomeraceae* sp. 1 subclade of the *N. plukenetiae sensu lato* group (GenBank accession number JX276891), providing further evidence that these AMF may be core members of the switchgrass microbiome. Finally, Gosling et al. (66) discussed the lack of ecological information about Paraglomerales as inspiration for their survey of this order across England. They found that Paraglomerales AMF were widely dispersed across agroecosystems but were more common in organically managed soils than conventionally managed soils. We expect that the widespread use of primers that are unbiased in their amplification of AMF rDNA (67) and resolution to the level of ASV (68) should promote greater inclusion and tractability of previously overlooked clades.

### AMF biogeography characterized by high beta diversity.

Similarly to previous work (e.g., reference 69), we documented high ASV beta diversity among our three sites. It is unclear to what extent AMF distribution is shaped by dispersal limitation versus environmental filtering. In our study, evidence for the former was the greater number of shared ASVs between the two closest sites (Oregon and Hancock) compared to those between the more distant adjacent sites (Hancock and Rhinelander), and the complete lack of shared ASVs between the sites at opposite ends of Wisconsin. However, the within-site correlation between community dissimilarity and soil N suggests that environmental filtering was also important in shaping AMF structure, and closer sites may have been more similar in soil variables important in filtering AMF. Using restriction fragment length polymorphism of AMF SSU rDNA, Hazard et al. (70) found that local environment, and not geographical distance, determined distribution of AMF in Ireland. If the rate of evolution in a population is greater than the rate of dispersal, the use of ASVs may provide fine enough taxonomic resolution to observe the evolution of population structure distinct from gene flow. Indeed, we do not know the time scales over which an AMF genotype can disperse 350 km, the approximate distance between our furthest sites, versus the rate of evolutionary change in internal transcribed spacer (ITS) rDNA that would result in different ASVs. More importantly, it remains to be determined if the genotypes represented by rDNA ASVs have genetic differences in other regions of the genome, with concomitant impacts on ecosystem functioning and switchgrass performance.

### Conclusions.

Four years of nitrogen addition at levels meant to replace harvested plant tissue N had no consistent effect on AMF composition or diversity. Across three Wisconsin sites, different factors were associated with switchgrass yield, highlighting the context dependency of switchgrass production for bioenergy. The ASVs elucidated in this study theoretically represent exact, living genotypes present in switchgrass soils. In the absence of taxonomic consensus about AMF species delimitations and, therefore, the full extent of intraspecific functional diversity (71), ASVs serve as consistent sequence identifiers for future studies seeking to understand AMF functioning and biogeography, as well as for bioprospecting of regionally adapted AMF inocula. To build a more predictive “mycorrhizal technology” for sustainable agricultural intensification of switchgrass (6), future research should evaluate more directly how specific AMF genotypes affect ecosystem functioning via soil biodiversity manipulation experiments (57).

## MATERIALS AND METHODS

### Sites and experimental design.

The U.S. Department of Energy’s Great Lakes Bioenergy Research Center designed a marginal lands experiment to test the efficacy of growing perennial biofuel feedstock crops on abandoned agricultural fields or land deemed unsuitable for high-productivity agriculture. This study focused on three Wisconsin sites: Rhinelander in northern Wisconsin, Hancock in the central part of the state, and Oregon in the south (see Fig. S1 in the supplemental material). The coordinates, soil texture, and mean soil physicochemical properties of the three sites are presented in Table S1 in the supplemental material; for a comprehensive assessment of the soils at each site, see Kasmerchak and Schaetzl (72), and for information on the ecology and land-use history of each site, see https://lter.kbs.msu.edu/research/long-term-experiments/marginal-land-experiment/. Monoculture plots of switchgrass (*Panicum virgatum* var. ‘Cave-in-Rock’) measuring 19.5 × 12.2 m were sown with seed in 2013. In 2015, N amendments were randomly applied to the plots in a paired experimental design, with half receiving no N amendment (control) and the other half receiving 56 kg · ha^−1^ N (amended) applied annually as granular ammonium nitrate. Thus, the experimental units in this study were the paired half-plots (19.5 × 6.1 m). The Rhinelander and Oregon sites each consisted of four replicates per treatment while the Hancock site consisted of three replicates per treatment (*n* = 11).

### Soil sampling and switchgrass yield.

In August 2018, a sliding-hammer soil corer fitted with a 5-cm diameter head was used to extract soil cores to a depth of 15 cm. Three soil cores spaced 1.8 m from the edge of the plot and 1 m from each other were collected in every experimental unit (Fig. S1). The cores were placed into Whirl-Pak bags and transported in an iced cooler to a −20°C freezer. Within 8 h, the soil samples were transferred to a −80°C freezer for storage prior to sieving. The soil samples were thawed in a refrigerator at 4°C overnight and manually disintegrated through a 2-mm sieve. The sieved soil was returned to the Whirl-Pak bags and preserved at −80°C until DNA extraction. Switchgrass yields were determined in November 2018 by harvesting switchgrass with a combine, leaving 15.25-cm residual stubble. Following switchgrass harvest, soil cores from each experimental unit were taken to a depth of 25 cm and analyzed for pH, phosphorus levels, and cation exchange capacity by the University of Wisconsin Soil and Forage Laboratory. Preserved soil samples from August were analyzed with a FlashEA 1112 flash combustion analyzer for total carbon and nitrogen determination.

### DNA extraction, amplification, and sequencing.

DNA was extracted from approximately 250 mg of soil from each sample with a DNeasy PowerSoil kit (catalog no. 12888-100; Qiagen) according to the manufacturer’s instructions. DNA concentration was measured with a Thermo Scientific NanoDrop 1000 spectrophotometer to determine dilution ratios for PCR. Extracts were amplified using the AMF-specific wobble primers wSSUmCf (5′-TAT YGY TCT TNA ACG AGG AAT C-3′) and wLSUmBr (5′-AAC ACT CGC AYA YAT GYT AGA-3′), which span an rDNA fragment consisting of the partial small-subunit locus (pSSU), whole internal transcribed spacer (ITS) region including the 5.8S locus, and partial large subunit locus (pLSU) (41, 67). Dried, 12-nmol RxnReady primer pools (forward and reverse primers premixed) were manufactured by Integrated DNA Technologies according to PacBio Sequel specifications, namely, high-performance liquid chromatography (HPLC) purification and the addition of a 5′ NH_4_-C_6_ block and universal sequence using PacBio-specific wSSUmCf (/5AmMC6/GCA GTC GAA CAT GTA GCT GAC TCA GGT CAC TAT YGY TCT TNA ACG AGG AAT C) and PacBio-specific wLSUmBr (/5AmMC6/TGG ATC ACT TGT GCA AGC ATC ACA TCG TAG AAC ACT CGC AYA YAT GYT AGA). For more information on the primer-specific requirements for successful PacBio sequencing, see https://www.pacb.com/wp-content/uploads/Procedure-Checklist-Preparing-SMRTbell-Libraries-using-PacBio-Barcoded-Universal-Primers-for-Multiplexing-Amplicons.pdf.

PCRs were prepared using the Phusion high-fidelity PCR kit from New England Biolabs (catalog no. E0553L) in 25-μl reaction mixtures, as follows: 1 μl of template DNA at a concentration of 0.1 to 40 ng · μl^−1^, 2.5 μl 10 μM primers, 0.5 μl 10 mM deoxynucleoside triphosphates (dNTPs), 0.25 μl Phusion DNA polymerase, 5 μl 5× Phusion HF buffer, and 15.75 μl water. PCRs were run on an Eppendorf Mastercycler Pro S thermal cycler using the parameters specified by Schlaeppi et al. (41), as follows: 2 min initial denaturation at 98°C, 40 cycles of 10-s denaturation at 98°C, 30 s annealing at 60°C, and 1 min elongation at 72°C, with a final elongation of 10 min at 72°C. Of the 66 soil samples, 48 (at least two samples per experimental unit) successfully amplified and were selected for sequencing. Samples were submitted to the University of Wisconsin Biotechnology Center and purified using AMPure XP beads, ligated with universal hairpin adapters for circular sequencing, and barcoded with sample-specific primers. The 48 samples were pooled based on molarity and sequenced on one PacBio Sequel SMRT cell with Sequel sequencing kit v3.0 chemistry.

### Bioinformatics.

PacBio Sequel subreads were demultiplexed and assembled into circular consensus sequences (CCS) with SMRT Analysis v6.0.0.47841 (*lima* v1.7.0 and *pbccs* v3.4.1). Sequences with fewer than five passes and a minimum predicted accuracy of <0.9 were removed, resulting in a prefiltering error rate comparable to that of Illumina MiSeq (41). CCS BAM files were converted to FASTQ format and their quality scores changed to the conventional 0 to 41 system using BBMap v38.50 (73). CCS were clustered into ASVs with DADA2 v1.14 (68, 74) according to the workflow and recommendations of the “DADA2 + PacBio” tutorial (68) (available at https://benjjneb.github.io/LRASManuscript/LRASms_fecal.html) on a MacBook Pro (early 2011 model) using R v3.6.0 (75). The resulting pSSU-ITS-pLSU ASVs were separated by rDNA locus using ITSx v1.1.2 (76) implemented in the R package *rITSx* v0.0.3 (77). A multilocus phylogenetic tree of complete ITS and partial LSU sequences was constructed with Tree-Based Alignment Selector (T-BAS) v2.1 on DeCIFR from North Carolina State University’s Center for Integrated Fungal Research (78) (accessible via https://decifr.hpc.ncsu.edu/) using auto values of the MAFFT algorithm, the GTRGAMMA substitution model, 100 bootstrap replicates, and *de novo* RAxML placement. ASVs with ambiguous rDNA loci according to ITSx or falling outside the Glomeromycotina according to the kingdom-wide phylogenetic tree were excluded from further analysis. Preliminary taxonomic assignments were made using DADA2’s implementation of the naive Bayesian classifier method from Wang et al. (79) and the UNITE v8.0 ITS fungal taxonomic reference (80, 81).

Two phylogenetic trees specific to AMF were subsequently constructed—one covering the entire Glomeromycotina subphylum and the other limited to the order Paraglomerales—to assign taxonomy and resolve undescribed clades. We manually assembled an LSU rDNA reference database representing 4 of 4 orders, 12 of 12 families, 40 of 41 genera, and 100 of 334 species currently described in Glomeromycotina (82, 83), including NCBI-designated type-material sequences from 14 species and rediscovered, undesignated type-material sequences from 48 other species (see Note S2 in the supplemental material). A further 38 species were represented by non-type material sequences. In total, 436 ASVs, 282 AMF reference sequences, and two type material sequences from Mucoromycotina as an outgroup were used in constructing the phylogenetic trees. We aligned our sequences using the E-INS-i iterative refinement method in MAFFT v7 (84) and conducted phylogenetic analysis with RAxML v8.2.11 (85) using the GTRGMMA substitution model and 100 bootstrap replicates. The best-fitting phylogenetic trees were visualized and annotated in iTOL v4 (86).

### Data analysis.

All analyses and statistical tests were conducted in R v3.6.0. Agronomic and soil data were averaged across replicates within each experimental unit, and ASV abundance was summed across replicates within each experimental unit. For each experimental unit, we calculated the following metrics of AMF alpha biodiversity using *vegan* v2.5.5 (87) or *picante* v1.8 (88): richness, Shannon diversity index, Simpson’s diversity index, Pielou’s evenness index, Faith’s phylogenetic diversity index, and phylogenetic mean pairwise distance (89). We tested the effect of N amendment on switchgrass yield and soil variables using random-intercept linear mixed effects models with lme4 v1.1.21 (90), specifying N treatment and site as interacting fixed effects and paired plot as a random grouping effect. To test for correlations between switchgrass yield and soil variables, we fitted separate random-intercept linear mixed effects models for each site to avoid issues of multicollinearity, specifying the soil variables and N treatment as fixed effects and paired plot as a random grouping effect. To determine the effects of N amendment on AMF community composition, we first visualized ASV UniFrac beta diversity with nonmetric multidimensional scaling (NMDS) ordination using *phyloseq* v1.28.0 (91). We then conducted a permutational multivariate analysis of variance (PERMANOVA) on UniFrac dissimilarity with *vegan*, specifying N treatment as the predictor and constraining permutations by site. We also tested for correlations between dissimilarity matrices of AMF community and abiotic soil variables using Mantel tests with *vegan*, again constraining permutations by site. Finally, we conducted an indicator species analysis on sites and N treatment using *indicspecies* v1.7.8 (92).

We visually checked assumptions of normally distributed errors and homogeneity of variance and transformed variables when appropriate to meet model assumptions. For linear mixed-effects models, we used *lmerTest* v3.1.0 (93) to calculate type III ANOVA *P* values via Satterthwaite’s degrees of freedom method. Statistical significance was determined by comparison to the Bonferroni critical value of *P < *0.002. For *post hoc* analyses, we used *lsmeans* v2.30.0 (94) to obtain least-square means and pairwise linear contrast Tukey-adjusted *P* values via the Kenward-Roger degrees of freedom method. We used *MuMIn* v1.43.15 (95) to calculate model conditional coefficients of determination (*R_c_^2^*). For the Mantel tests, statistical significance was set to the Bonferroni critical value of *P < *0.008.

### Data availability.

Raw CCS reads are accessible via National Center for Biotechnology Information (NCBI) under the Sequence Read Archive (SRA) BioProject accession number PRJNA590305 (BioSample accession numbers SAMN13324198 to SAMN13324245). Amplicon sequence variants are available through NCBI under GenBank accession numbers MT765295 to MT765730. Reproducible bioinformatics scripts and R markdown documents are available at https://github.com/aldendirks/amf_metabarcoding.

## Supplementary Material

Supplemental file 1
