# Supplementary material for: Community Structure of Arbuscular Mycorrhizal Fungi in Soils of Switchgrass Harvested for Bioenergy
Source: Appl Environ Microbiol. 2020 Sep 17;86(19):e00880-20. doi: 10.1128/AEM.00880-20 (PMC7499029; doi:10.1128/AEM.00880-20)
Supplement: Supplemental file 1 [file AEM.00880-20-s0001.pdf]

Supplemental Information

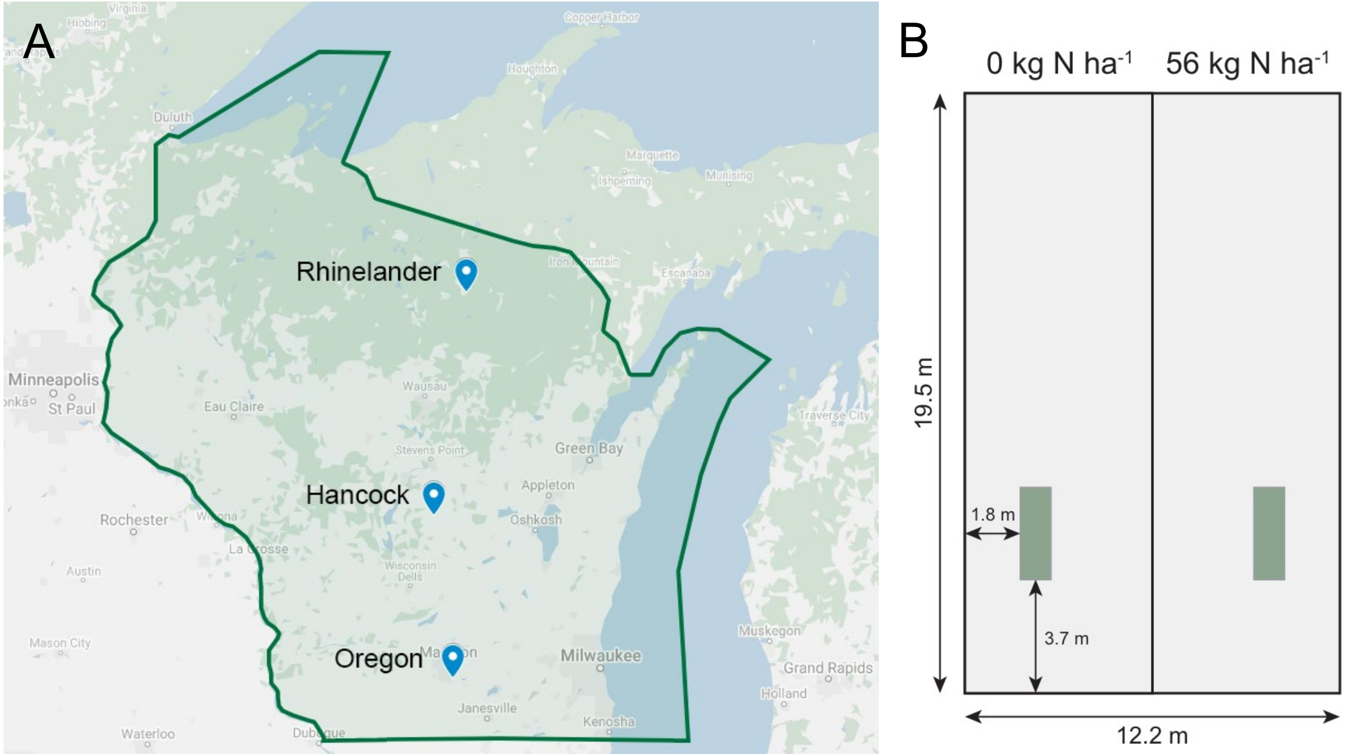

Figure S1. A) Map of Wisconsin labelled with the three Wisconsin Marginal Lands Experiment sites (Google Maps). B) Representative diagram of the experimental units. Switchgrass plots were divided in half, one of which received no N amendment (0 kg N ha^{-1}) and the other 56 kg ha^{-1} N annually. Rhinelander and Oregon consisted of four replicates each and Hancock consisted of three replicates. Soil samples for arbuscular mycorrhizal fungal metabarcoding and soil C and N analyses were collected from the areas indicated by green rectangles.

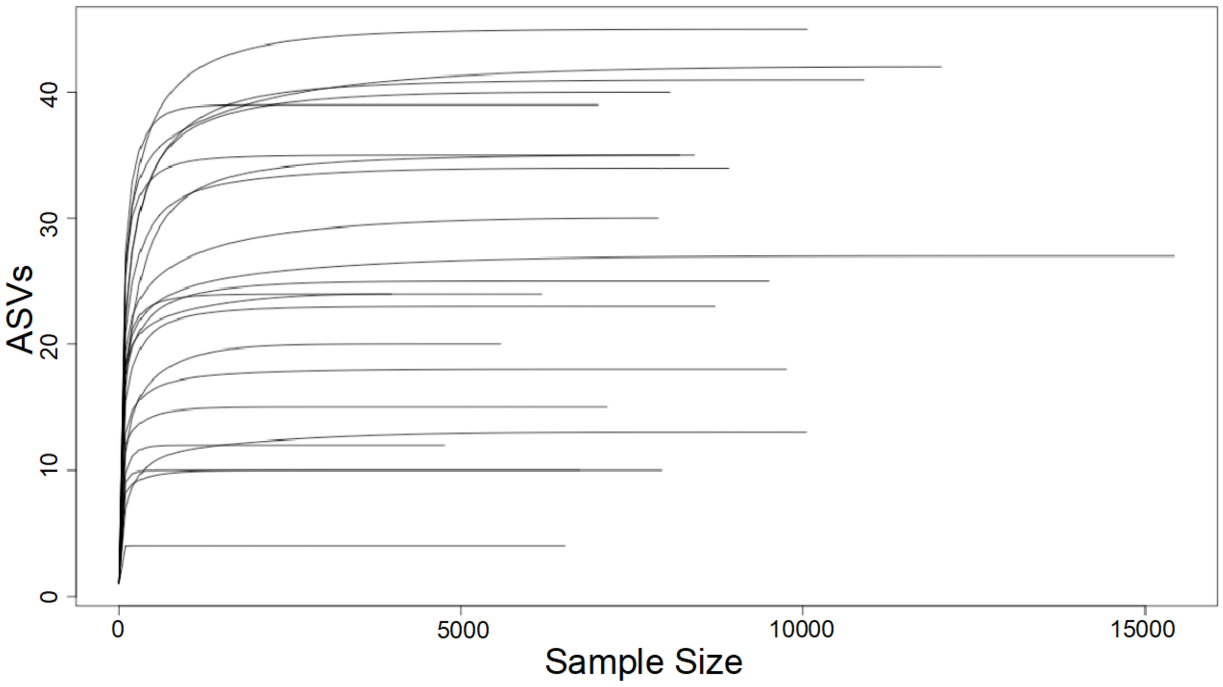

Figure S2. To determine the completeness of our sampling efforts, we plotted rarefaction curves for the 22 experimental units analyzed in this study using the “rarecurve” function from the package *vegan* in R. ASV richness reached clear asymptotes for all experimental units, indicating that there was sufficient sequencing depth to recover the majority of AMF ASVs.

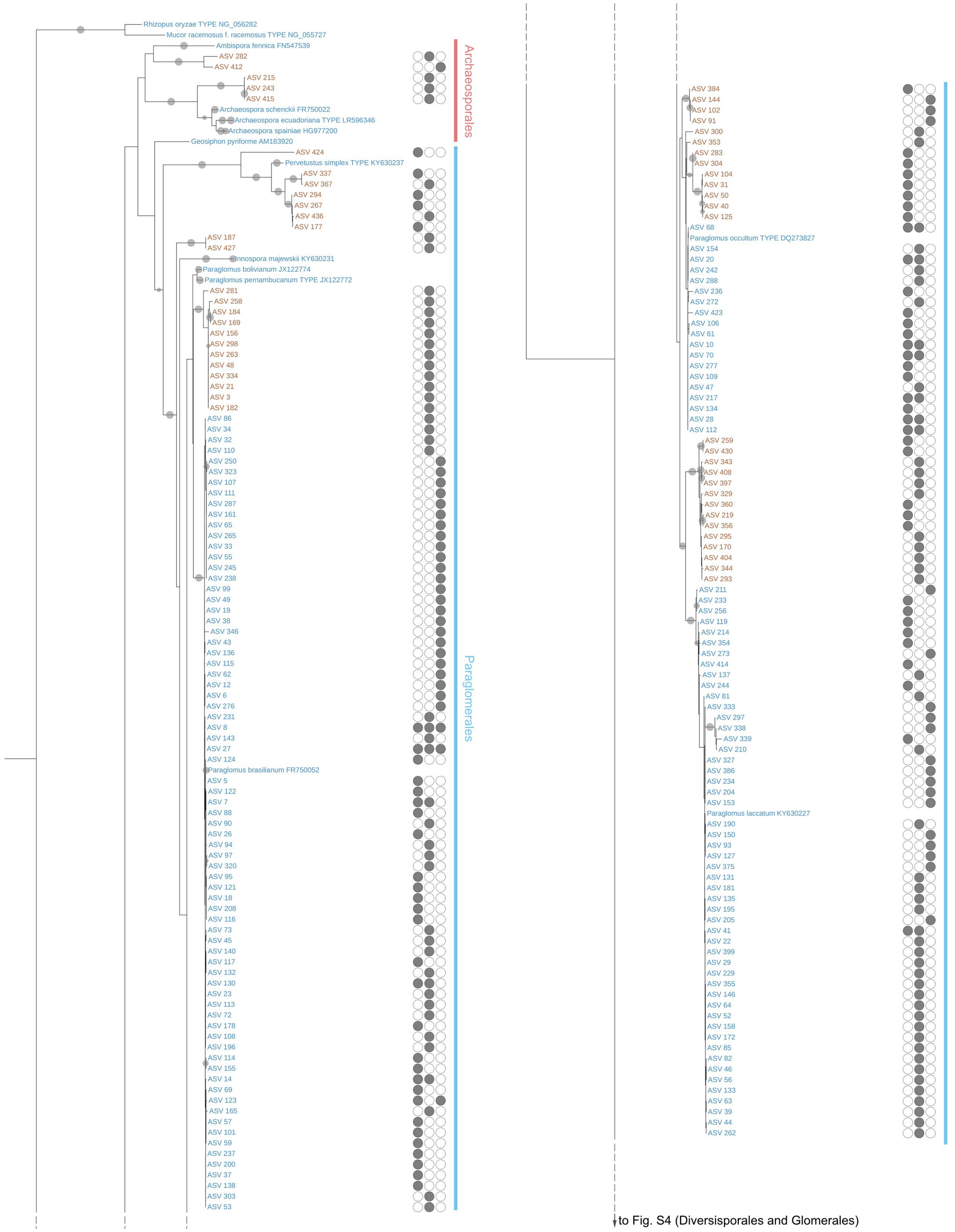

Rhizopus oryzae TYPE NG_056282
Mucor racemosus f. racemosus TYPE NG_055727
Ambispora fennica FN547539
ASV 282
ASV 412
ASV 215
ASV 243
ASV 415
Archaeospora schenckii FR750022
Archaeospora ecuadoriana TYPE LR596346
Archaeospora spainiae HG977200
Geosiphon pyriforme AM183920
ASV 424
Pervetustus simplex TYPE KY630237
ASV 337
ASV 367
ASV 294
ASV 267
ASV 436
ASV 177
ASV 187
ASV 427
Innospora majewskii KY630231
Paraglomus bolivianum JX122774
Paraglomus pernambucanum TYPE JX122772
Archaeosporales
Paraglomerales
Paraglomus brasilianum FR750052
Paraglomus occultum TYPE DQ273827
Paraglomus laccatum KY630227
to Fig. S4 (Diversisporales and Glomerales)

Figures S3. Phylogenetic tree of Archaeosporales and Paraglomerales reference taxa and ASVs recovered in this study. The three circles adjacent to the taxon name correspond to site occurrence, with a filled circle indicating presence at that site. From left to right, the circles correspond to Hancock, Oregon, and Rhinelander. Bootstrap support ≥ .75 is indicated by grey circles on the branches.

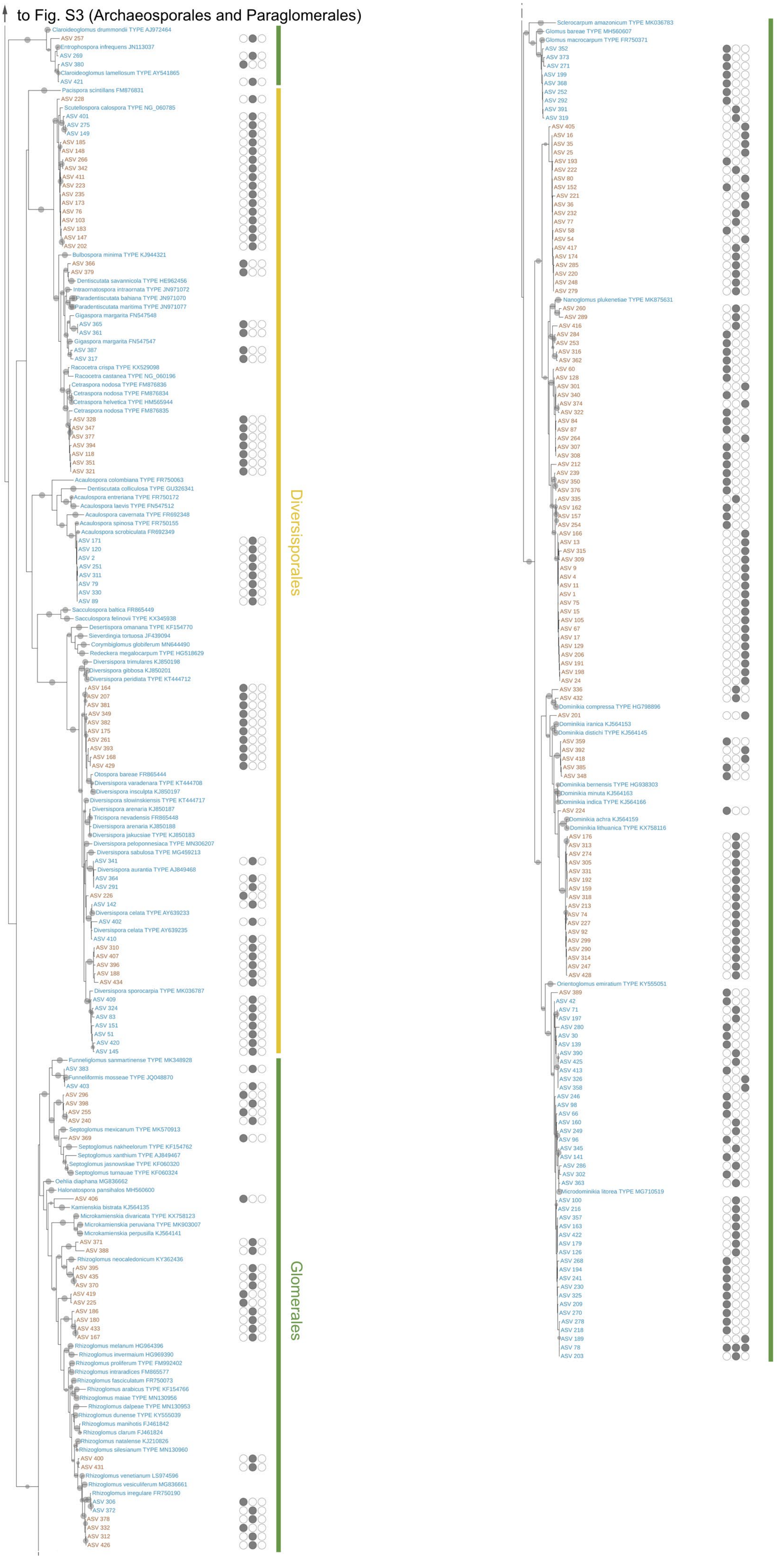

to Fig. S3 (Archaeosporales and Paraglomerales)
Claroideoglomus drummondii TYPE AJ972464
ASV 257
Entrophospora infrequens JN113037
ASV 269
ASV 380
Claroideoglomus lamellosum TYPE AY541865
ASV 421
Pacispora scintillans FM876831
ASV 228
Scutellospora calospora TYPE NG_060785
ASV 401
ASV 275
ASV 149
ASV 185
ASV 148
ASV 266
ASV 342
ASV 411
ASV 223
ASV 235
ASV 173
ASV 76
ASV 103
ASV 183
ASV 147
ASV 202
Bulbospora minima TYPE KJ944321
ASV 366
ASV 379
Dentiscutata savannicola TYPE HE962456
Intraornatospora intraornata TYPE JN971072
Paradentiscutata bahiana TYPE JN971070
Paradentiscutata maritima TYPE JN971077
Gigaspora margarita FN547548
ASV 365
ASV 361
Gigaspora margarita FN547547
ASV 387
ASV 317
Racocetra crispa TYPE KX529098
Racocetra castanea TYPE NG_060196
Cetraspora nodosa TYPE FM876836
Cetraspora nodosa TYPE FM876834
Cetraspora helvetica TYPE HM565944
Cetraspora nodosa TYPE FM876835
ASV 328
ASV 347
ASV 377
ASV 394
ASV 118
ASV 351
ASV 321
Acaulospora colombiana TYPE FR750063
Dentiscutata colliculosa TYPE GU326341
Acaulospora entreriana TYPE FR750172
Acaulospora laevis TYPE FN547512
Acaulospora cavernata TYPE FR692348
Acaulospora spinosa TYPE FR750155
Acaulospora scrobiculata TYPE FR692349
ASV 171
ASV 120
ASV 2
ASV 251
ASV 311
ASV 79
ASV 330
ASV 89
Sacculospora baltica FR865449
Sacculospora felinovii TYPE KX345938
Desertispora omanana TYPE KF154770
Sieverdingia tortuosa JF439094
Corymbiglomus globiferum MN644490
Redeckera megalocarpum TYPE HG518629
Diversispora trimulares KJ850198
Diversispora gibbosa KJ850201
Diversispora peridiata TYPE KT444712
ASV 164
ASV 207
ASV 381
ASV 349
ASV 382
ASV 175
ASV 261
ASV 393
ASV 168
ASV 429
Otospora bareae FR865444
Diversispora varadenara TYPE KT444708
Diversispora insculpta KJ850197
Diversispora slowinskiensis TYPE KT444717
Diversispora arenaria KJ850187
Tricispora nevadensis FR865448
Diversispora arenaria KJ850188
Diversispora jakucsiae TYPE KJ850183
Diversispora peloponnesiaca TYPE MN306207
Diversispora sabulosa TYPE MG459213
ASV 341
Diversispora aurantia TYPE AJ849468
ASV 364
ASV 291
ASV 226
ASV 142
Diversispora celata TYPE AY639233
ASV 402
Diversispora celata TYPE AY639235
ASV 410
ASV 310
ASV 407
ASV 396
ASV 188
ASV 434
Diversispora sporocarpia TYPE MK036787
ASV 409
ASV 324
ASV 83
ASV 151
ASV 51
ASV 420
ASV 145
Funneliglomus sanmartinense TYPE MK348928
ASV 383
Funneliformis mosseae TYPE JQ048870
ASV 403
ASV 296
ASV 398
ASV 255
ASV 240
Septoglomus mexicanum TYPE MK570913
ASV 369
Septoglomus nakheelorum TYPE KF154762
Septoglomus xanthium TYPE AJ849467
Septoglomus jasnowskae TYPE KF060320
Septoglomus turnauae TYPE KF060324
Oehlia diaphana MG836662
Halonatospora pansihalos MH560600
ASV 406
Kamienskia bistrata KJ564135
Microkamienskia divaricata TYPE KX758123
Microkamienskia peruviana TYPE MK903007
Microkamienskia perpusilla KJ564141
ASV 371
ASV 388
Rhizoglomus neocaledonicum KY362436
ASV 395
ASV 435
ASV 370
ASV 419
ASV 225
ASV 186
ASV 180
ASV 433
ASV 167
Rhizoglomus melanum HG964396
Rhizoglomus invermaium HG969390
Rhizoglomus proliferum TYPE FM992402
Rhizoglomus intraradices FM865577
Rhizoglomus fasciculatum FR750073
Rhizoglomus arabicus TYPE KF154766
Rhizoglomus maiae TYPE MN130956
Rhizoglomus dalpeae TYPE MN130953
Rhizoglomus dunense TYPE KY555039
Rhizoglomus manihotis FJ461842
Rhizoglomus clarum FJ461824
Rhizoglomus natalense KJ210826
Rhizoglomus silesianum TYPE MN130960
ASV 400
ASV 431
Rhizoglomus venetianum LS974596
Rhizoglomus vesiculiferum MG836661
Rhizoglomus irregulare FR750190
ASV 306
ASV 372
ASV 378
ASV 332
ASV 312
ASV 426
Diversisporales
Glomerales
Sclerocarpum amazonicum TYPE MK036783
Glomus bareae TYPE MH560607
Glomus macrocarpum TYPE FR750371
ASV 352
ASV 373
ASV 271
ASV 199
ASV 368
ASV 252
ASV 292
ASV 391
ASV 319
ASV 405
ASV 16
ASV 35
ASV 25
ASV 193
ASV 222
ASV 80
ASV 152
ASV 221
ASV 36
ASV 232
ASV 77
ASV 58
ASV 54
ASV 417
ASV 174
ASV 285
ASV 220
ASV 248
ASV 279
Nanoglomus plukenetiae TYPE MK875631
ASV 260
ASV 289
ASV 416
ASV 284
ASV 253
ASV 316
ASV 362
ASV 60
ASV 128
ASV 301
ASV 340
ASV 374
ASV 322
ASV 84
ASV 87
ASV 264
ASV 307
ASV 308
ASV 212
ASV 239
ASV 350
ASV 376
ASV 335
ASV 162
ASV 157
ASV 254
ASV 166
ASV 13
ASV 315
ASV 309
ASV 9
ASV 4
ASV 11
ASV 1
ASV 75
ASV 15
ASV 105
ASV 67
ASV 17
ASV 129
ASV 206
ASV 191
ASV 198
ASV 24
ASV 336
ASV 432
Dominikia compressa TYPE HG798896
ASV 201
Dominikia iranica KJ564153
Dominikia disticha TYPE KJ564145
ASV 359
ASV 392
ASV 418
ASV 385
ASV 348
Dominikia bernensis TYPE HG938303
Dominikia minuta KJ564163
Dominikia indica TYPE KJ564166
ASV 224
Dominikia achra KJ564159
Dominikia lithuanica TYPE KX758116
ASV 176
ASV 313
ASV 274
ASV 305
ASV 331
ASV 192
ASV 159
ASV 318
ASV 213
ASV 74
ASV 227
ASV 92
ASV 299
ASV 290
ASV 314
ASV 247
ASV 428
Orientoglomus emiratium TYPE KY555051
ASV 389
ASV 42
ASV 71
ASV 197
ASV 280
ASV 30
ASV 139
ASV 390
ASV 425
ASV 413
ASV 326
ASV 358
ASV 246
ASV 98
ASV 66
ASV 160
ASV 249
ASV 96
ASV 345
ASV 141
ASV 286
ASV 302
ASV 363
Microdominikia litorea TYPE MG710519
ASV 100
ASV 216
ASV 357
ASV 163
ASV 422
ASV 179
ASV 126
ASV 268
ASV 194
ASV 241
ASV 230
ASV 325
ASV 209
ASV 270
ASV 278
ASV 218
ASV 189
ASV 78
ASV 203
Glomerales

Figure S4. Phylogenetic tree of Diversisporales and Glomerales reference taxa and ASVs recovered in this study. The three circles adjacent to the taxon name correspond to site occurrence, with a filled circle indicating presence at that site. From left to right, the circles correspond to Hancock, Oregon, and Rhinelander. Bootstrap support ≥ .75 is indicated by grey circles on the branches.

Table S1. Coordinates, texture, and mean physiochemical properties of the surface soils of the Wisconsin Marginal Lands Experiment sites (1).

**Site**			**Soil properties**						
**Name**	**Latitude**	**Longitude**	**Texture**	**C:N**	**pH**	**Sand (%)**	**Silt (%)**	**Clay (%)**	**Bulk density (g cm^{-3})**
Rhinelander	45° 39' 56.16" N	89° 13' 4.8" W	Sandy loam	15.9	5.7	59.1	25.8	15.1	1.35 ± 0.20
Hancock	44° 7' 9.84" N	89° 32' 1.68" W	Loamy sand	12.7	6.3	87.6	5.9	6.5	1.44 ± 0.03
Oregon	42° 57' 57.96" N	89° 21' 21.96" W	Silt loam	10.2	6.9	9.1	74.8	16.1	1.11 ± 0.37

Table S2. Sequences retained through each of the steps of the *DADA2* bioinformatics pipeline. "CCS" (circular consensus sequences) is the number of sequences that were generated with a minimum of five passes during PacBio sequencing. "Primers" is the number of sequences that contained the AMF-specific pSSU-ITS-pLSU primer sequences. "Filtered" is the number of sequences that had a quality score greater than or equal to three, expected error less than or equal to two, and sequence length between 1000 and 1600 base pairs. "Denoised" is the number of sequences that were inferred as ASVs according to the PacBio error-learning algorithm of *DADA2*. "Non-chimeric" is the number of sequences remaining after removing chimeric ones. Finally, "Retained" is the fraction of sequences retained at the end of the pipeline (non-chimeric sequences divided by circular consensus sequences). The bottom two rows of the table show the averages and sums of each column.

**Site**	**Block**	**Subsample**	**Nitrogen treatment**	**CCS**	**Primers**	**Filtered**	**Denoised**	**Non-chimeric**	**Retained**
Rhinelander	R1	B	amended	7625	7409	5042	3699	3699	0.485115
Rhinelander	R1	C	amended	9580	9298	6808	5812	5812	0.606681
Rhinelander	R1	E	control	7971	7614	4782	2247	2247	0.281897
Rhinelander	R1	F	control	4284	4172	2898	1922	1922	0.448646
Rhinelander	R1	G	control	4735	4575	3140	2042	2042	0.431257
Rhinelander	R2	A	amended	7443	7276	4375	2963	2963	0.398092
Rhinelander	R2	B	amended	8115	7821	5104	3571	3571	0.440049
Rhinelander	R2	E	control	9348	9146	5310	3042	3042	0.325417
Rhinelander	R2	F	control	9673	9477	5837	4452	4452	0.46025
Rhinelander	R2	G	control	1760	1695	936	443	443	0.251705
Rhinelander	R3	A	amended	6316	6106	3402	2088	2088	0.330589
Rhinelander	R3	B	amended	9902	9652	5301	2749	2749	0.277621
Rhinelander	R3	C	amended	10029	9739	6414	5223	5223	0.52079
Rhinelander	R3	E	control	3183	2982	2004	1327	1327	0.416902
Rhinelander	R3	G	control	8523	8336	6413	6037	5812	0.68192
Rhinelander	R4	B	amended	8769	8525	6340	5633	5215	0.594709
Rhinelander	R4	C	amended	9470	9231	5992	4543	4543	0.479725

Rhinelander	R4	E	control	9121	8959	6265	5257	5257	0.576362
Rhinelander	R4	G	control	3950	3856	2581	1482	1482	0.37519
Hancock	R2	A	amended	8132	7927	5518	5080	4417	0.543163
Hancock	R2	C	amended	7178	6982	5265	4868	4539	0.632349
Hancock	R2	E	control	6446	6301	4655	3603	3475	0.539094
Hancock	R2	G	control	6444	6233	4414	3658	3530	0.547796
Hancock	R3	A	amended	2947	2872	2098	1843	1708	0.579572
Hancock	R3	C	amended	6515	6322	4133	3265	3118	0.478588
Hancock	R3	E	control	10195	9970	6448	5294	5029	0.493281
Hancock	R3	G	control	9905	9625	5975	4210	3920	0.39576
Hancock	R4	A	amended	8902	8689	6532	5877	5234	0.587958
Hancock	R4	B	amended	10559	10230	3739	3083	2963	0.280614
Hancock	R4	C	amended	2314	2205	1632	1409	1294	0.559205
Hancock	R4	E	control	10105	9826	6431	4565	4073	0.403068
Hancock	R4	G	control	10458	10225	7867	7417	6868	0.656722
Oregon	R1	A	amended	9277	8639	5974	4896	4884	0.526463
Oregon	R1	B	amended	12384	11687	9144	8523	8268	0.667636
Oregon	R1	E	control	190	179	124	53	53	0.278947
Oregon	R1	G	control	11320	10980	7380	5998	5556	0.490813
Oregon	R2	A	amended	11048	10729	8677	8599	8541	0.773081
Oregon	R2	B	amended	11959	11690	8900	8636	6895	0.576553
Oregon	R2	E	control	10351	10141	7989	7643	6206	0.599556
Oregon	R2	G	control	9263	9002	6796	5924	5817	0.627982
Oregon	R3	A	amended	8433	8221	5918	5240	4940	0.585794
Oregon	R3	B	amended	6795	6607	4580	3726	3474	0.511258
Oregon	R3	E	control	9754	9563	7049	6366	5213	0.534447
Oregon	R3	G	control	5337	5217	3587	2677	2677	0.501593
Oregon	R4	A	amended	10589	10279	7036	5880	5338	0.504108
Oregon	R4	C	amended	10295	10028	6376	5123	4746	0.461
Oregon	R4	E	control	6785	6608	4714	4033	4031	0.594105
Oregon	R4	F	control	134	130	77	4	4	0.029851
**SUM**	———	———	———	**373811**	**362976**	**247972**	**202025**	**190700**	**NA**
**AVERAGE**	———	———	———	**7788**	**7562**	**5166**	**4209**	**3973**	**0.486318**

Note S1

PacBio is criticized for low sequencing depth and a high error rate, which may deter its broader use in the study of AMF (2). While PacBio has a lower sequencing depth than Illumina sequencing technologies and therefore may not describe microbial communities as fully, this is not a problem for relatively low-diversity groups like Glomeromycotina (3–4). Furthermore, sequencing depth may be a moot point with increased access to PacBio Sequel 2, which generates up to 4 million reads compared to Sequel's 500,000. In regard to high error rates, the circularization and multiple sequencing passes of individual DNA molecules result in an error rate comparable to – or even less than – other leading platforms (5). In conjunction with the use of error-learning bioinformatics algorithms like *DADA2*, the average number of erroneous bases is less than one for every 2000 nucleotides, resulting in single-nucleotide resolution for medium-length amplicons like those employed in this study (6). The length of amplicons generated with PacBio have the additional benefit of primarily capturing living organisms in the community as the majority of relic DNA from dead organisms consists of fragments < 200 bases in length (7).

Note S2

In our curation of a Glomeromycotina LSU database for phylogenetics, we discovered 351 sequences that were derived from type cultures but not labelled with the "type_material" source key in GenBank. Most undesignated type-material sequences were discovered by searching for accessions referenced in primary literature describing new species of Glomeromycotina fungi; a number of others were found by searching for sequences not labelled as type material but containing the query "epitype", "holotype", "isotype", or "sp. nov.". Although of the highest importance, these sequences were therefore not retrievable as type material via Entrez Direct (EDirect), the suite of command-line utilities for interfacing with National Center for Biotechnology Information databases (8). Given that GenBank contains numerous misidentified DNA sequences, a label to distinguish confidently identified sequences from those that might be erroneous is a necessity for sequence-based identification (9). Barring contamination, sequences from type material are always correct according to the logic of taxonomy. Thus, it is essential that GenBank sequences from type material are accurately labelled as such so that they can be discerned from all other sequences, whose identities are hypotheses or best guesses of varying reliability. We notified staff at NCBI and authors of theses sequences, who are working to correct the metadata of these accessions.

For public sequence repositories to serve the wider research community, mycologists must take care to ensure and submit high-quality metadata (9). Poorly annotated sequences propagate through UNITE and SILVA, resulting in erroneous and imprecise taxonomic assignments of environmental amplicon sequences. As one example, four pSSU-ITS-pLSU type-material sequences were generated and deposited in GenBank for the description of *Diversispora jakucsiae*: KJ850181- KJ850185 (10). In GenBank, these accessions are labelled as *Diversispora*

sp. JB-2014 both in the title and as the source/organism. There is no “type_material” key allowing these sequences to be downloaded as type sequence with EDirect. The UNITE species hypothesis linked to these sequences is labelled as *Diversispora* sp. with no indication that these sequences are from type material. The linked SILVA LSU entry also lists these sequences as *Diversispora* sp., makes no indication of type status, and, furthermore, erroneously categorizes *Diversispora* sp. in Mortierellales (Mucoromycotina). Thus, during taxonomic assignment, amplicons belonging to *Diversispora jakucsiae* would be imprecisely identified as *Diversispora* sp. in the case of UNITE or be placed in a completely wrong subphylum if using SILVA. Even when the GenBank source is accurately listed to species but the title is listed at a broader taxonomic rank, as is the case with the type-material sequences for *Pervetustus simplex* (KY630235-KY630251; 11), the UNITE species hypothesis is labelled simply as Paraglomeromycetes. This incomplete classification explains why none of the ASVs belonging to *Pervetustaceae* in our study were identified as such when referencing UNITE. Incomplete database curation prevents full insight into community composition and hinders a broader understanding AMF ecology and biogeography from environmental sequences.

References

1. Kasmerchak CS, Schaetzl R. 2018. Soils of the GLBRC Marginal Land Experiment (MLE) sites. Kellogg Biological Station Long-term Ecological Research Special Publication. Zenodo. http://doi.org/10.5281/zenodo.2578238.
2. Kennedy PG, Cline LC, Song Z. 2018. Probing promise versus performance in longer read fungal metabarcoding. New Phytol 217:973–976.
3. Tedersoo L, Tooming-Klunderud A, Anslan S. 2018. PacBio metabarcoding of Fungi and other eukaryotes: Errors, biases and perspectives. New Phytol 217:1370–1385.
4. Vasar M, Andreson R, Davison J, Jairus T, Moora M, Remm M, Young JPW, Zobel M, Öpik M. 2017. Increased sequencing depth does not increase captured diversity of arbuscular mycorrhizal fungi. Mycorrhiza 27:761–773.
5. Schlaeppi K, Bender SF, Mascher F, Russo G, Patrignani A, Camenzind T, Hempel S, Rillig MC, van der Heijden MGA. 2016. High-resolution community profiling of arbuscular mycorrhizal fungi. New Phytol 212:780–791.
6. Callahan BJ, Wong J, Heiner C, Oh S, Theriot CM, Gulati AS, Mcgill SK, Dougherty MK. 2019. High-throughput amplicon sequencing of the full-length 16S rRNA gene with single-nucleotide resolution. Nucleic Acids Res 47:1–12.
7. Nilsson RH, Anslan S, Bahram M, Wurzbacher C, Baldrian P, Tedersoo L. 2019. Mycobiome diversity: High-throughput sequencing and identification of fungi. Nat Rev Microbiol 17:95–109.
8. Federhen S. 2015. Type material in the NCBI Taxonomy Database. Nucleic Acids Res 43:D1086–D1098.

9. Hofstetter V, Buyck B, Eyssartier G, Schnee S, Gindro K. 2019. The unbearable lightness of sequenced-based identification. Fungal Divers 3:243–284.

10. Balázs TK, Błaszkowski J, Chwat G, Góralska A, Gáspár BK, Lukács AF, Kovács GM. 2015. Spore-based study of arbuscular mycorrhizal fungi of semiarid sandy areas in Hungary, with *Diversispora jakucsiae* sp. nov. Mycol Prog 14:1–11.

11. Błaszkowski J, Kozłowska A, Crossay T, Symanczik S, Al-Yahya'ei MN. 2017. A new family, *Pervetustaceae* with a new genus, *Pervetustus*, and *P. simplex* sp. nov. (Paraglomerales), and a new genus, *Innospora* with *I. majewskii* comb. nov. (*Paraglomeraceae*) in the Glomeromycotina. Nov Hedwigia 105:397–410.
